# Supplementary material for: Binding of heterochromatin protein Rhino to a subset of piRNA clusters depends on a combination of two histone marks
Source: Nat Struct Mol Biol. 2025 Jun 17;32(8):1517–27. doi: 10.1038/s41594-025-01584-8 (PMC12350163; doi:10.1038/s41594-025-01584-8)
Supplement: Supplementary file 1 — Supplementary Notes 1 and 2. [file 41594_2025_1584_MOESM1_ESM.pdf]

nature structural & molecular biology

Supplementary information https://doi.org/10.1038/s41594-025-01584-8

# Binding of heterochromatin protein Rhino to a subset of piRNA clusters depends on a combination of two histone marks

In the format provided by the authors and unedited

# Table of Contents

**Supplementary Notes**
Supplementary Note 1
Supplementary Note 2

**Supplementary Note 1 (related to Extended Data Fig. 1): PRC2 components recovered in screen**

Depletion of *caf1-55* resulted in rudimentary ovaries rendering the analyses of the germline TE expression difficult. Knockdowns of *esc*, *esc1* and *Su(z)12* did not result in sterility of the F1 generation contrary to expected; knockdown of *E(z)* resulted in F1 sterility in the mini screen. This suggests that knockdown efficiencies were not optimal and/or a redundant role of the homologues esc and esc1 and resulted in different effects on TE de-repression; different TE families appear to have different sensitivity to low levels of PRC2.

**Supplementary Note 2 (related to Fig. 2): Antibody cross-reactivity**

Early on in our study we noticed that regions with very high H3K27me3 levels also displayed some H3K9me3 signal. This was particularly evident when plotting H3K27me3 against H3K9me3 signal across genome-wide 1 kb bins (**Fig. N1**), where surprisingly, high H3K27me3 in euchromatic regions is reflected by elevated H3K9me3 signal. A similar observation was made genome-wide (**Extended Data Fig. 3a**), where regions with very high H3K27me3 level also show distinctive H3K9me3 peaks. Since the H3K27me3 methyltransferase E(z) is not expected to be able to write both marks, we concluded that the H3K9me3 antibodies likely displayed a weak cross-reactivity with H3K27me3.

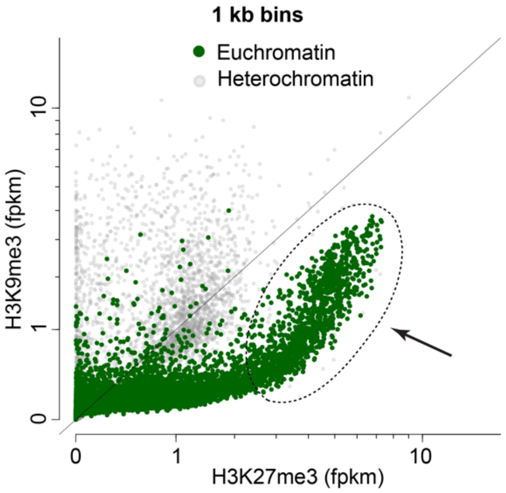

**Fig. N1:** Scatter plot showing H3K27me3 and H3K9me3 levels from CUT&RUN with antibodies in 1 kb bins separated into euchromatin (green) and heterochromatin (grey).

This cross-reactivity was further supported in S2 cells using an *E(z)* knockdown. Globally, S2 cells displayed a marked reduction in H3K27me3 signal following *siE(z)* treatment, as expected, whereas H3K9me3 signal was largely unaltered (**Fig. N2**). However, specific regions with extremely high H3K27me3 signal lost their corresponding H3K9me3 peak following E(z) depletion (**Fig. N2**, yellow areas). Notably, these regions were not bound by the CBX1 chromodomain, a known H3K9me3 reader, and are therefore unlikely to reflect true H3K9me3 signal. Hence, we concluded that H3K9me3 antibodies display a weak cross-reactivity with H3K27me3.

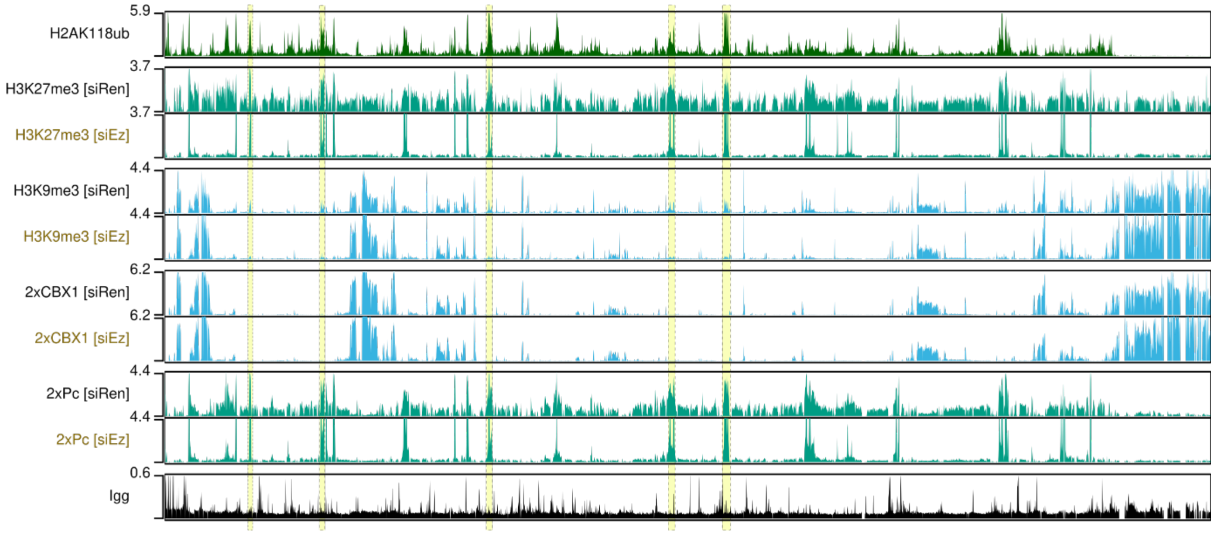

**Fig. N2:** UCSC genome browser tracks displaying H2AK118ub, H3K9me3 and H3K27me3 histone mark signal as well as the 2xCBX1 and 2xPc CD construct signal obtained from CUT&RUN upon the indicated knockdowns.

Although the H3K9me3 and H3K27me3 signals detected by the H3K9me3 antibody strongly differed in amplitude, the potential cross-reactivity made it difficult to evaluate how well H3K9me3 on its own was predictive for dual-strand cluster locations. For these analyses, we therefore opted to use HP1a binding as an alternative method to measure H3K9me3 in *Drosophila* ovaries (**Fig. 2d-f, Extended Data Fig. 3e**), and CD$^{2xCBX1}$ binding as a marker for H3K9me3 in S2 cells (**Extended Data Fig. 7**), ensuring that the results could be unambiguously interpreted.
