# Supplementary material for: Binding of heterochromatin protein Rhino to a subset of piRNA clusters depends on a combination of two histone marks
Source: Nat Struct Mol Biol. 2025 Jun 17;32(8):1517–27. doi: 10.1038/s41594-025-01584-8 (PMC12350163; doi:10.1038/s41594-025-01584-8)
Supplement: Supplementary file 2 — Reporting Summary [file 41594_2025_1584_MOESM2_ESM.pdf]

nature portfolio

Corresponding author(s): Gregory J Hannon, Benjamin Czech Nicholson, Emilie Brasset

Last updated by author(s): Apr 18, 2025

Reporting Summary

Nature Portfolio wishes to improve the reproducibility of the work that we publish. This form provides structure for consistency and transparency in reporting. For further information on Nature Portfolio policies, see our Editorial Policies and the Editorial Policy Checklist.

Statistics

For all statistical analyses, confirm that the following items are present in the figure legend, table legend, main text, or Methods section.

n/a	Confirmed	
☐	☒	The exact sample size (*n*) for each experimental group/condition, given as a discrete number and unit of measurement
☐	☒	A statement on whether measurements were taken from distinct samples or whether the same sample was measured repeatedly
☐	☒	The statistical test(s) used AND whether they are one- or two-sided<br>*Only common tests should be described solely by name; describe more complex techniques in the Methods section.*
☒	☐	A description of all covariates tested
☐	☒	A description of any assumptions or corrections, such as tests of normality and adjustment for multiple comparisons
☐	☒	A full description of the statistical parameters including central tendency (e.g. means) or other basic estimates (e.g. regression coefficient) AND variation (e.g. standard deviation) or associated estimates of uncertainty (e.g. confidence intervals)
☐	☒	For null hypothesis testing, the test statistic (e.g. *F*, *t*, *r*) with confidence intervals, effect sizes, degrees of freedom and *P* value noted<br>*Give P values as exact values whenever suitable.*
☒	☐	For Bayesian analysis, information on the choice of priors and Markov chain Monte Carlo settings
☒	☐	For hierarchical and complex designs, identification of the appropriate level for tests and full reporting of outcomes
☒	☐	Estimates of effect sizes (e.g. Cohen's *d*, Pearson's *r*), indicating how they were calculated

Our web collection on statistics for biologists contains articles on many of the points above.

Software and code

Policy information about availability of computer code

Data collection	No software was used for data collection.
Data analysis	HTS data processing: FastQC v0.11.8, Trim Galore! v0.6.4 or v0.6.6, STAR v2.7.3a, bowtie v1.2.3, bowtie2 v2.4.2, Picard tools v2.21.2<br>Differential expression analysis: featureCounts v1.5.3, DESeq2 v1.26.0<br>Genome browser tracks: deepTools v3.3.2 or v3.5.0<br>Peak calling and analysis: MACS2 v2.2.7.1, DiffBind v3.8.4<br>Structure modelling: UCSF Chimera v1.16, ZDOCK v3.0.2<br>Statistical analysis and visualisation: R v3.6.2, pheatmap v1.0.12, sinkr v0.6, eulerr package (v7.0.2)<br>Image analysis: Fiji/ImageJ software v1.54p

For manuscripts utilizing custom algorithms or software that are central to the research but not yet described in published literature, software must be made available to editors and reviewers. We strongly encourage code deposition in a community repository (e.g. GitHub). See the Nature Portfolio guidelines for submitting code & software for further information.

Data

Policy information about availability of data

All manuscripts must include a data availability statement. This statement should provide the following information, where applicable:

- Accession codes, unique identifiers, or web links for publicly available datasets
- A description of any restrictions on data availability
- For clinical datasets or third party data, please ensure that the statement adheres to our policy

Sequencing data generated in this study has been deposited to the GEO under accession (GSE247156). The following data were retrieved from GEO: RNA-seq, sRNA-seq and ChIP-seq data for Rhi and Kipf were downloaded from the GEO (accession GSE202468),. HP1a ChIP-seq data was downloaded from GEO (GSE140542). Rhi ChIP-seq samples from control, Rhi, and Moon knockout ovaries input samples were downloaded from GEO (accession GSE97719), sRNA-seq data for Drosophila species (GSE225888). The dm6 genome assembly was downloaded from the UCSC genome browser and the following assemblies were downloaded from RefSeq: GCF_003285975, GCF_003286155, GCF_016746395, and GCF_016746365. Source data are provided with this paper.

Research involving human participants, their data, or biological material

Policy information about studies with human participants or human data. See also policy information about sex, gender (identity/presentation), and sexual orientation and race, ethnicity and racism.

Reporting on sex and gender	N/A
Reporting on race, ethnicity, or other socially relevant groupings	N/A
Population characteristics	N/A
Recruitment	N/A
Ethics oversight	N/A

Note that full information on the approval of the study protocol must also be provided in the manuscript.

Field-specific reporting

Please select the one below that is the best fit for your research. If you are not sure, read the appropriate sections before making your selection.

☒ Life sciences ☐ Behavioural & social sciences ☐ Ecological, evolutionary & environmental sciences

For a reference copy of the document with all sections, see nature.com/documents/nr-reporting-summary-flat.pdf

Life sciences study design

All studies must disclose on these points even when the disclosure is negative.

Sample size	As the number of flies to be used in the experiments was not a limiting factor, no statistical power analyses were used to predetermine sample sizes. Sample sizes were chosen as large as possible while still practically feasible in term of data collection. All experiments in this work build on established experimental schemes in the piRNA and Drosophila genetics fields. Adequate statistics has been applied throughout the manuscript in order to make sure that the observed effects are significant given the reported sample size.
Data exclusions	Two CUT&RUN samples were excluded from the analysis due to failing quality control, as stated in the manuscript. These samples are marked as failed but nevertheless made available.
Replication	Experiments were independently repeated, the number of replicates are presented in the figure legends and/or methods.
Randomization	Randomization was not relevant to this study. All flies were from similar genetic background and groups for statistical comparisons were constructed based on treatment (knockdown target).
Blinding	No investigator blinding was applied during data acquisition or analyses as the data was mostly analyzed in bulk by (blind) scripts such as for NGS or FISH quantification or blinding was not desirable for data presentation.

Reporting for specific materials, systems and methods

We require information from authors about some types of materials, experimental systems and methods used in many studies. Here, indicate whether each material, system or method listed is relevant to your study. If you are not sure if a list item applies to your research, read the appropriate section before selecting a response.

Materials & experimental systems

n/a	Involved in the study
☐	☒ Antibodies
☐	☒ Eukaryotic cell lines
☒	☐ Palaeontology and archaeology
☐	☒ Animals and other organisms
☒	☐ Clinical data
☒	☐ Dual use research of concern
☒	☐ Plants

Methods

n/a	Involved in the study
☐	☒ ChIP-seq
☒	☐ Flow cytometry
☒	☐ MRI-based neuroimaging

Antibodies

Antibodies used

anti-Rhino polyclonal, Rabbit pAB ,Eurogentec, Mohn et al., 2014, ChIP: 5µl; IF 1:1000
anti-Histone H3K9me3 antibody, Rabbit pAB , Active motif #39161, ChIP: 5µl; CUT&RUN 1:20
anti-Histone H3K27me3 antibody, Rabbit mAB, Cell Signaling Technology #9733S, CUT&RUN 1:20
anti-Histone H3K27me3 antibody, Rabbit pAB, Millipore #07-449, ChIP 5µl
anti-H2AK119Ub antibody, Rabbit mAB, Cell Signaling Technology #8240, CUT&RUN 1:20
anti-FLAG M2 antibody, Mouse mAB, Sigma Aldrich #F1804, CUT&RUN 1:20; WB: 1:2500
anti-Kipf A96 antibody, Mouse mAB, Baumgartner et al., 2022, Brennecke lab, WB: 1:200
anti-alphaTubulin antibody, Rabbit pAB, Abcam #ab18251, WB: 1:5000
anti-Rabbit IgG IRDye 680LT, Goat pAB, Licor #926-68021, WB 1:10000
anti-Mouse IgG IRDye 800CW, Goat pAB, Licor #926-32210, WB 1:5000

Validation

All commercially available antibodies were validated by the manufacturers. Non-commercially available antibodies used in this study have been validated in previous publications (cited in this manuscript).

All validation statements, including citation for commercial antibodies can be found on the manufacturers' websites:
anti-Histone H3K9me3: https://www.activemotif.com/catalog/details/39161/histone-h3-trimethyl-lys9-antibody-pab
anti-Histone H3K27me3 (Cell signalling):: https://www.cellsignal.com/products/primary-antibodies/tri-methyl-histone-h3-lys27-c36b11-rabbit-mab/9733
anti-Hitsone H3K27me3 (Milipore):https://www.merckmillipore.com/DE/en/product/Anti-trimethyl-Histone-H3-Lys27-Antibody,MM_NF-07-449
anti-H2AK119Ub: https://www.cellsignal.com/products/primary-antibodies/ubiquityl-histone-h2a-lys119-d27c4-xp-rabbit-mab/8240
anti-FLAG M2: https://www.sigmaaldrich.com/DE/en/product/sigma/f1804
anti-Kipf : The antoby was validated in Baugmarten et al., 2022
anti-aplhaTubulin: https://www.abcam.com/en-us/products/primary-antibodies/alpha-tubulin-antibody-microtubule-marker-ab18251

Eukaryotic cell lines

Policy information about cell lines and Sex and Gender in Research

Cell line source(s)

Used cell line in this study: Drosophila melanogaster Schneider 2 cells (S2 cells). S2 cells were purchased from ThermoFisher (#R69007).

Authentication

Drosophila S2 cells were not authenticated, however, next-generation sequencing confirmed their identify as Drosophila cells.

Mycoplasma contamination

S2 cells were regularly checked for mycoplasma by an in-house facility. The cell line used was mycoplasma negative.

Commonly misidentified lines
(See ICLAC register)

N/A

Animals and other research organisms

Policy information about studies involving animals; ARRIVE guidelines recommended for reporting animal research, and Sex and Gender in Research

Laboratory animals

This study exclusively involved work with Drosophila melanogaster, a standard invertebrate model organism that does not underlie any ethical restrictions. Standard laboratory procedures have been applied throughout the study. Drosophila strains used in this study are described in the Methods section. Adult females used for ovary dissections were aged 2–6 days as stated in the Methods section.

Wild animals

The study did not involve wild animals.

Reporting on sex

The study focused on the ovarian function of E(z) and Rhino and therefore used only females.

Field-collected samples	The study did not involve samples collected from the wild.
Ethics oversight	No ethical approval or guidance were required as the study used Drosophila.

Note that full information on the approval of the study protocol must also be provided in the manuscript.

Plants

Seed stocks	N/A
Novel plant genotypes	N/A
Authentication	N/A

ChIP-seq

Data deposition

☒ Confirm that both raw and final processed data have been deposited in a public database such as GEO.

☒ Confirm that you have deposited or provided access to graph files (e.g. BED files) for the called peaks.

Data access links<br>*May remain private before publication.*	https://www.ncbi.nlm.nih.gov/geo/query/acc.cgi?acc=GSE247156
Files in database submission	H3K27me3.nos-E(z).1 [ChIP-seq]<br>H3K27me3.nos-E(z).2 [ChIP-seq]<br>H3K27me3.nos-E(z).3 [ChIP-seq]<br>H3K27me3.nos-white.1 [ChIP-seq]<br>H3K27me3.nos-white.2 [ChIP-seq]<br>H3K27me3.nos-white.3 [ChIP-seq]<br>H3K9me3.nos-E(z).1 [ChIP-seq]<br>H3K9me3.nos-E(z).2 [ChIP-seq]<br>H3K9me3.nos-white.1 [ChIP-seq]<br>H3K9me3.nos-white.2 [ChIP-seq]<br>input.nos-E(z).1 [ChIP-seq]<br>input.nos-E(z).2 [ChIP-seq]<br>input.nos-white.1 [ChIP-seq]<br>input.nos-white.2 [ChIP-seq]<br>Rhino.nos-E(z).1 [ChIP-seq]<br>Rhino.nos-E(z).2 [ChIP-seq]<br>Rhino.nos-white.1 [ChIP-seq]<br>Rhino.nos-white.2 [ChIP-seq]<br>H3K27me3.nos-Kipf.1.2 [ChIP-seq]<br>H3K27me3.nos-Kipf.2.2 [ChIP-seq]<br>H3K27me3.nos-White.1.2 [ChIP-seq]<br>H3K27me3.nos-White.2.2 [ChIP-seq]<br>H3K27me3.nos-White.3.2 [ChIP-seq]<br>input.nos-Kipf.1.2 [ChIP-seq]<br>input.nos-White.1.2 [ChIP-seq]
Genome browser session<br>(e.g. UCSC)	UCSC Genome Browser-compatible BigWig (.bw) files are included in the GEO submission.

Methodology

Replicates	2-3 biological replicates were performed per sample.
Sequencing depth	Name Total Unique Length Type<br>H3K27me3.nos-E(z).1 14556897.00 2861898.00 100 single<br>H3K27me3.nos-E(z).2 32064702.00 20754163.00 100 single<br>H3K27me3.nos-E(z).3 35148363.00 22601795.00 100 single<br>H3K27me3.nos-white.1 16004969.00 3838357.00 100 single<br>H3K27me3.nos-white.2 21280635.00 10288146.00 100 single<br>H3K27me3.nos-white.3 31984551.00 23322089.00 100 single<br>H3K9me3.nos-E(z).1 36960490.00 11622760.00 100 paired-end

H3K9me3.nos-E(z).2 30653102.00 11795243.00 100 paired-end
H3K9me3.nos-white.1 36524968.00 12982482.00 100 paired-end
H3K9me3.nos-white.2 39929034.00 11551417.00 100 paired-end
input.nos-E(z).1 20141957.00 17048831.00 100 single
input.nos-E(z).2 32559246.00 27390817.00 100 paired-end
input.nos-white.1 20323312.00 16403946.00 100 single
input.nos-white.2 31386746.00 26555400.00 100 paired-end
Rhino.nos-E(z).1 39255318.00 11981984.00 100 paired-end
Rhino.nos-E(z).2 36020694.00 19117573.00 100 paired-end
Rhino.nos-white.1 38668340.00 28323873.00 100 paired-end
Rhino.nos-white.2 47157362.00 28907720.00 100 paired-end
H3K27me3_Wh.1.2 83310948.00 50015531.00 100 paired-end
H3K27me3_Wh.2.2 95069608.00 55097575.00 100 paired-end
H3K27me3_Wh.3.2 146792032.00 81935056.00 100 paired-end
H3K27me3_Kip.1.2 71411850.00 40050953.00 100 paired-end
H3K27me3_Kip.2.2 83203520.00 46435848.00 100 paired-end
input_White.1.2 89380548.00 73077128.00 100 paired-end
Input_Kipferl.1.2 130128714.00 106785429.00 100 paired-end

Antibodies

ChIPseq_anti Rhino samples: Anti-Rhino polyclonal antibody produced in Rabbit (Mohn et al 2014)
ChIPseq_anti H3K27me3 samples: anti-H3K27me3 (Millipore 07-449)
ChIPseq_anti H3K9me3 samples: anti-H3K9me3 (active motif 39161)

Peak calling parameters

Peaks were called using MACS2 (v2.2.7.1) to capture narrow (-q 0.05 -g dm) and broad peaks (-q 0.05 -g dm –broad –broad-cutoff 0.1). Only uniquely mapped reads were used for the peak calling. As a control we used Input for each condition.

Data quality

Visual inspection of data in the genome browser to confirm that the ChIPseq signal in wildtype accumulates as expected based on previous literature

Software

ChIP-seq reads were aligned to the dm6 genome using Bowtie2 (v2.4.2), with the alignment process set to report at most one hit for each read. In case of alignments with the same MAPQ score, the best alignment was randomly selected from among those equally scored alignments. Peaks were called using MACS2 (v2.2.7.1) to capture narrow (-q 0.05 -g dm) and broad peaks (-q 0.05 -g dm --broad --broad-cutoff 0.1). Only uniquely mapped reads were used for the peak calling. As a control we used Input for each condition.
