# Supplementary figures and images for: Binding of heterochromatin protein Rhino to a subset of piRNA clusters depends on a combination of two histone marks
Source: Nat Struct Mol Biol. 2025 Jun 17;32(8):1517–27. doi: 10.1038/s41594-025-01584-8 (PMC12350163; doi:10.1038/s41594-025-01584-8)

related to Extended Data Figure 6i

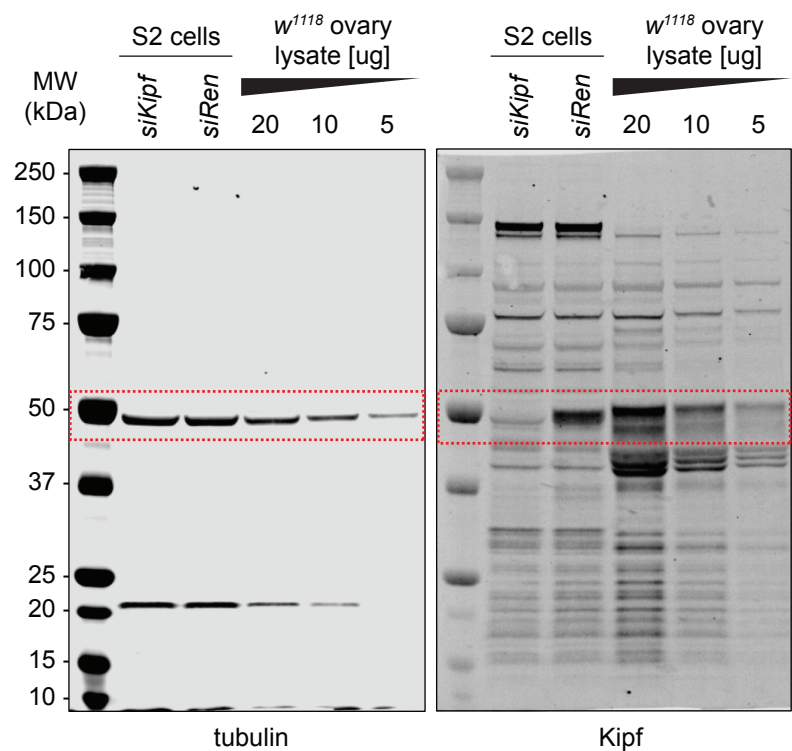

related to Extended Data Figure 6j

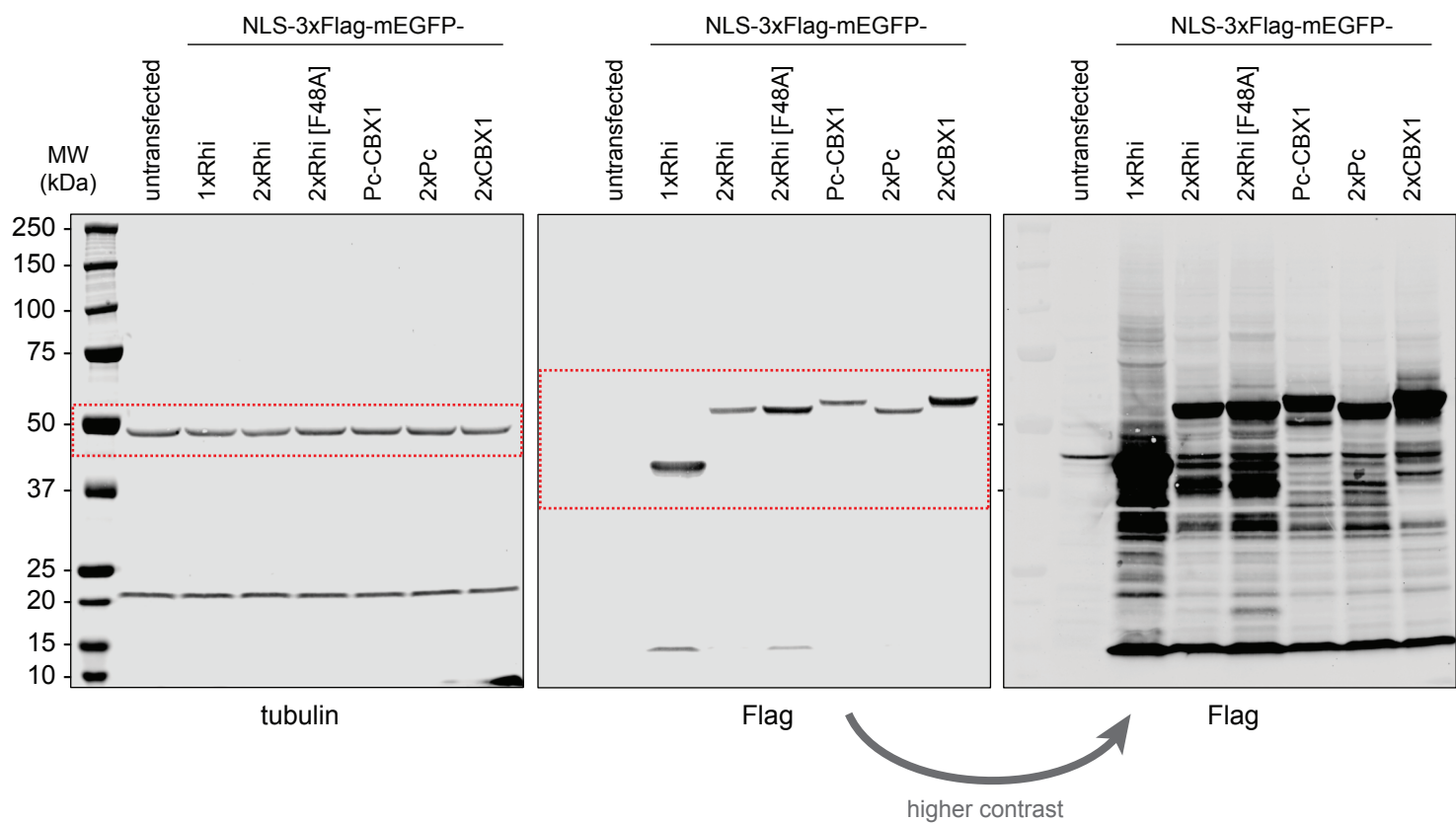

Supplement: Supplementary file 15 — Uncropped western blots. [file 41594_2025_1584_MOESM15_ESM.pdf]
